# Radiology subspecialisation in Africa: A review of the current status

**DOI:** 10.4102/sajr.v25i1.2168

**Published:** 2021-08-30

**Authors:** Efosa P. Iyawe, Bukunmi M. Idowu, Olasubomi J. Omoleye

**Affiliations:** 1 College of Medicine, University of Ibadan, Ibadan, Oyo State, Nigeria; 2 Department of Radiology, Union Diagnostics and Clinical Services PLC, Yaba, Lagos State, Nigeria; 3 Department of General Medicine, LouisMed Hospital and Fertility Centre, Lekki Phase 1, Lagos State, Nigeria

**Keywords:** Africa, curriculum, education, radiology, specialist training, subspecialisation, web-based learning

## Abstract

**Background:**

Radiology subspecialisation is well-established in much of Europe, North America, and Australasia. It is a natural evolution of the radiology speciality catalysed by multiple factors.

**Objectives:**

The aim of this article is to analyse and provide an overview of the current status of radiology subspecialisation in African countries.

**Methods:**

We reviewed English-language articles, reports, and other documents on radiology specialisation and subspecialisation in Africa.

**Results:**

There are 54 sovereign countries in Africa (discounting disputed territories). Eighteen African countries with well-established radiology residency training were assessed for the availability of formal subspecialisation training locally. Eight (Egypt, Ethiopia, Kenya, Morocco, Nigeria, South Africa, Tanzania, and Tunisia) out of the 18 countries have local subspecialist training programmes. Data and/or information on subspecialisation were unavailable for three (Algeria, Libya, and Senegal) of the 18 countries. Paediatric Radiology (Ethiopia, Nigeria, South Africa, Tunisia) and Interventional Radiology (Egypt, Kenya, South Africa, Tanzania) were the most frequently available subspecialist training programmes. Except Tanzania, all the countries with subspecialisation training programmes have ≥ 100 radiologists in their workforce.

**Conclusion:**

There is limited availability of subspecialist radiology training programmes in African countries. Alternative models of subspecialist radiology training are suggested to address this deficit.

## Epigraph

‘A radiologist is a clinician who has sacrificed one of the greatest glories of the practice of medicine and its greatest responsibility – the daily contact with the ill and their families – in order to concentrate more on the essence of our profession, the pathology of the living. This he sees through the medium of shadows which has left him open to the charge of not quite being a clinician. But shadows after all are real. Seeing is done with the mind. The camera does not see, it records. The clinician at the bedside sees the patient and imagines the lesion. The radiologist sees the lesion and imagines the patient’.

[Harry Z. Mellins, MD (1921 – 2009); former Professor of Radiology, Harvard Medical School]

## Introduction

Clinical and non-clinical knowledge, procedural expertise, computer literacy/proficiency in information technology, knowledge of diagnostics, and non-interpretive skills are an integral part of postgraduate training in radiology:^1,2,3^

A Radiologist is a specialist medical doctor who has had postgraduate training in performing and interpreting diagnostic imaging tests, and carrying out interventional procedures or treatments, using X-ray, ultrasound, and Magnetic Resonance Imaging equipment.^4,5^

A medical speciality is a branch/subdivision/subset of medicine that focuses on or is devoted to a specific or defined category of patients, illnesses or disorders, expertise or skills, or philosophy or theory.^6^ Radiology, Internal Medicine, Paediatrics, Otorhinolaryngology (ORL), Ophthalmology, Surgery, and so on, are examples of medical specialities. A physician who has specialised in a medical speciality is a specialist. In contrast, a generalist’s competencies, proficiency, interests, and skills/expertise are diverse, eclectic, comprehensive, and unspecialised. In other words, a generalist maintains a broad scope of practice.^7,8^ A general radiologist is one who has undergone a radiology residency without completing a fellowship or any other subspeciality training.^9^

A subspeciality is a narrow field of professional knowledge, skills, expertise, and focus within a broader medical speciality:^10^

Subspecialisation entails the devotion of intellectual energies to understand more and more about a narrower aspect of a speciality. It provides an intense exposure to a subspeciality area allowing a focused development of clinical and surgical skills related to that subspeciality area.^11,12^

Subspecialisation requires knowledge, expertise, and practice beyond that of a specialisation. Specific personnel, equipment, technology, dedicated curriculum, accredited training centres, logbooks, exposure to complex cases, high case volumes, subspecialist international journals, international subspecialist societies/associations, and relevant scientific breakthroughs in the field are the sine qua non of subspecialisation.^13^

In essence, a radiology subspeciality should have a unique body of knowledge that cannot be subsumed under general radiology, a peculiar applicability distinct from general radiology, an evidence of improved patient care because of subspecialist input, a structured formal training, and accruable benefits which outweigh any negative impact on the extant general radiology or other radiology subspeciality.^14^

Factors such as the expansive and multifaceted nature of radiology, rapid development of new imaging modalities, the need for subspecialist radiology interpretation for subspecialist referring physicians, more competitive job market, increased prospects of better remuneration, improved professional standing (status symbol), and so on, are the chief drivers of the quest for subspecialisation in radiology.^15^

Existing radiology subspecialities include system-based subspecialities (Cardiovascular Imaging, Chest Imaging, ORL/Dental Imaging, Breast Imaging, Musculoskeletal Imaging, Gastrointestinal Imaging, Uroradiology, Obstetric & Gynaecological Imaging, Neuroradiology); technique-based subspecialities (Nuclear Medicine/Radionuclide Radiology, Interventional Radiology [IR]); disease-based subspecialities (Oncological Imaging, Trauma Imaging, Emergency Radiology); age-based subspecialities (Paediatric Imaging); and emerging subspecialities (Global Radiology, Radiology Informatics, Forensic Radiology).^16^

In general, ‘radiology’ arrived in Africa, not too long after Roentgen discovered X-rays in 1895, when X-ray machines became available in South Africa (1896),^17^ Egypt (1906),^18^ Uganda (1907),^19^ Nigeria (1913),^20^ and so on. However, the commencement of radiology postgraduate residency and subspecialisation in African countries seems to have lagged behind the rest of the world.

An article published in 2019 reported that subspeciality training was available in 0%, 55%, and 74% of African, European, and Asian countries, respectively.^21^ The pros and cons of radiology subspecialisation had been discussed comprehensively by other authors.^9,22,23,24,25,26^

This research aims to investigate the current status of radiology subspecialisation training programmes in Africa and to identify the obstacles to the actualisation of this laudable aspiration. The focus is on the availability of local formal training programmes rather than the presence or absence of practising subspecialist radiologists in the health workforce of the countries evaluated.

## Methods

We searched the literature using Google search engine (primarily), Google Scholar, and African Journal Online. The keywords comprised the name of the countries together with the words radiology, specialisation, and subspecialisation in various combinations. The references cited in the retrieved articles provided additional information. The country reports published by RAD-AID International (https://rad-aid.org/resource-center/country-reports) were invaluable sources of data. The last search was performed on 20th April 2021. A few North African radiologists were also contacted by email to provide information about their countries, but we received a response from only one of them.

## Results

A summary of available subspecialist radiology training programmes in different African countries is presented in Table 1. Only those countries with well-established radiology residency training were assessed for the availability of formal subspecialisation training locally. Eight (Egypt, Ethiopia, Kenya, Morocco, Nigeria, South Africa, Tanzania, and Tunisia) out of the 18 African countries with well-established radiology residency training have local subspecialist training programmes. East Africa (three countries), North Africa (three countries), Southern Africa (one country), and West Africa (one country) make up the regional spread. Data/information on subspecialisation were unavailable for three of the 18 countries (Algeria, Libya, and Senegal). Except for Tanzania, all the countries with subspecialisation training programmes have ≥ 100 radiologists in their workforce.

**TABLE 1:** Current status of radiology subspecialisation in Africa.

Country	Population	Year estimated	Radiologist workforce	Year estimated	Radiology residency	Duration (years)	Qualification(s) awarded	Subspeciality radiology training program
Algeria^29,30^	43 851 044	2020	788	2012	Yes	4	NA	NA
Cameroon^31^	26 545 863	2020	50	2017	Yes	4	MD	None
CIV^32,33^	26 378 274	2020	60	2011	Yes	4	Diploma/Certificate of Specialized Studies	None
Egypt^29,30,34^	102 334 404	2020	1250	2012	Yes	5	MSc or MD or FEBR	IR; WI
Ethiopia^29,30^	114 963 588	2020	100	2012	Yes	4	MD	PR
Ghana^29,35^	31 072 940	2020	56	2020	Yes	5	FGCPS or FWACS or combination	None
Kenya^29,36^	53 771 296	2020	146	2020	Yes	6	MMed	IR
Libya^37^	6 871 292	2020	55	2017	Yes	NA	Libyan Fellowship Certificate/Medical Specialties Board Certificate	NA
Mauritius^38,39^	1 271 768	2020	54	2020	Yes	NA	MD	None
Morocco^40^	36 910 560	2020	338	2018	Yes	NA	MD	IR
Nigeria^20^	206 139 589	2020	355	2011	Yes	5	FWACS or FMCR or MD or combination	PR
Rwanda^29,41,42^	12 952 218	2020	11	2015	Yes	4	MMed	None
Senegal^29^	16 743 927	2020	NA	-	Yes	≥ 7	NA	NA
South Africa^29,43,44^	59 308 690	2020	650	2015	Yes	5	FCRadSA or MMed or combination	PR; IR
Sudan^45,46^	43 849 260	2020	30	2013	Yes	4	MD	None
Tanzania^29,47^	59 734 218	2020	60	2020	Yes	3	MMed	IR
Tunisia^48^	11 818 619	2020	750	2016	Yes	5	MD	IR; PR
Uganda^29,49^	45 741 007	2020	50	2019	Yes	3	MMed	None

Note: Current population estimates obtained from Worldometer elaboration of the latest United Nations data (https://www.worldometers.info/world-population/).

CIV, Cote D’Ivoire; FCRadSA, Fellow College of Radiology of South Africa; FEBR, Fellow Egyptian Board of Radiology; FGCPS, Fellow Ghana College of Physicians & Surgeons; FMCR, Fellow (of the) Medical College (in) Radiology (Nigeria); FWACS, Fellow West African College of Surgeons; IR, Interventional Radiology; MD, Doctor of Medicine; MMed, Master of Medicine; MSc, Master of Science; NA, Not Available; PR, Paediatric Radiology; WI, Women Imaging.

### Paediatric radiology

Paediatric radiology subspeciality training is available in South Africa, Ethiopia, and possibly, Tunisia.

#### South Africa

In South Africa, paediatric radiology subspeciality training began in 2009 at the Red Cross War Memorial Hospital in Cape Town. It is a 1-year programme after which successful candidates are awarded a postgraduate diploma in paediatric radiology by the University of Cape Town. Subspeciality training in paediatric imaging is also available at the Nelson Mandela Children’s Hospital in Johannesburg (opened officially in December 2016).^27^

Also in South Africa, the World Federation for Paediatric Imaging (WFPI), under the auspices of the William Shiels memorial foundation, offers a 3-month funded paediatric radiology observership in South Africa, at either the Red Cross War Memorial Children’s Hospital in Cape Town or the Nelson Mandela Children’s Hospital in Johannesburg. The first WFPI Observer/Fellow completed her Observership in 2018. The 2020 programme was shelved because of the COVID-19 pandemic.^28^

#### Ethiopia

Efforts to set up a paediatric radiology subspeciality training programme at the Department of Radiology, Addis Ababa University, Ethiopia commenced in 2012 as a 4-year outreach programme by paediatric radiology staff of the Children’s Hospital of Philadelphia (CHOP), Perelman School of Medicine, University of Pennsylvania, USA (CHOP Paediatric Radiology International Outreach Programme) in collaboration with the WFPI. The 2-year-long subspeciality fellowship programme finally took off in 2015 and has already produced two locally trained paediatric radiologists (graduated in 2017). There are three new trainees in the programme as of 2019.^50,51,52^

#### Tunisia

Professor Hassen El-Akeba Gharbi is a celebrated Tunisian paediatric radiologist and past president of the World Federation for Ultrasound in Medicine and Biology (WFUMB), who started a paediatric radiology department at the Children’s Hospital, Tunis, Tunisia, in 1970. He taught paediatric radiology at the hospital, and the programme still exists.^53^ Only the radiology department of the children’s hospital in Tunis can train paediatric radiologists (Prof. Ibtissem Bellagha, 2020, Personal email communication, 08 October)

#### Nigeria

A recent article from Nigeria indicates that paediatric radiology subspeciality training became available under the auspices of the West African Postgraduate Medical College in 2019. It appears that the programme is still nascent and the details are sketchy.^54^

#### Continental Professional Association/Society

The African Society of Paediatric Imaging (AfSPI) was formalised on 30th October 2012.^55^

### Interventional radiology

Subspeciality training in IR is available in Egypt, South Africa, Kenya, Tanzania, Tunisia, and Morocco.^29,56,57^

#### South Africa

Formal 1-year subspeciality training in IR was established at the University of the Orange Free State (UOFS), Bloemfontein, in 2002. A diploma in IR of the UOFS is conferred at the successful completion of the programme.^57^ More training centres may have been established in South Africa thereafter.

#### Egypt

In Egypt, there is no structured nationwide IR fellowship yet; however, IR subspeciality certification is currently organised via two pathways for doctors who have already completed their diagnostic radiology training. Firstly, there is institution-based training in which Fellows rotate through 4–5 self-selected training centres from a pool of 10 accredited training institutes/university hospitals. Secondly, candidates can apply to the Egyptian Board of Interventional Radiology (currently the only official IR specialty board certificate in Egypt) for a 2-year training program.^56,58,59^

#### Tanzania

The Tanzanian 2-year IR subspeciality curriculum took off in October 2019 at the Muhimbili National Hospital (MNH) in Dar es Salaam.^60^ The first class of three fellows is expected to graduate by September 2021 with a Master of Science in Interventional Radiology to be awarded by the Muhimbili University of Health and Allied Sciences (MUHAS). The Tanzanian IR project was midwifed by the RAD-AID International IR program in collaboration with academic institutions in the United States and Europe.^47,60,61^

#### Kenya

In June 2020, the University of Nairobi’s senate approved a 2-year IR fellowship domiciled at the university’s Department of Diagnostic Imaging and Radiation Medicine. The inaugural class of Kenyan Diagnostic radiologists would be enrolled later in 2020. This programme was established with the help of the University of North Carolina at Chapel Hill (Division of Vascular-Interventional Radiology & Division of Radiologic Science) and RAD-AID International.^62^

Finally, there is circumstantial evidence of IR subspeciality training in Morocco and Tunisia,^59^ but details of the programmes could not be obtained.

#### Continental Professional Association/Society

The Society of African Interventional Radiology and Endovascular Therapy (SAFIRE) is the continental professional association of interventional radiologists in Africa. Some of the North African countries are also members of the Pan Arab Interventional Radiology Society (PAIRS).

### Women’s imaging

There is anecdotal evidence of institution-based subspeciality training in Women’s Imaging at Cairo University in Egypt.

## Discussion

As stated at the introduction, a previous article published in 2019 reported inaccurately that subspeciality training was available in 0% of African countries. This misinformation is likely because of the general paucity of information on the evolution of radiology in African countries. As can be seen from our data, IR training had been available in South Africa since 2002. The quest for radiology subspecialisation seems to have accelerated in the last 5 years across the continent. In spite of this positive development, many hurdles still remain.

### Obstacles to the establishment of radiology subspecialisation in Africa

Africa, being a continent of mostly developing and underdeveloped countries, faces many challenges to the smooth implementation of radiology subspecialisation. Some of these obstacles include lack of funding, equipment and infrastructure deficit, unavailability of expertise, politics, emigration of radiologists, perfectionism, and so on.

#### Funding

Generally, the health sector is not adequately funded by many African governments and other stakeholders. The improvement of any field or speciality requires adequate funding for optimal growth and development. Sufficient funding allows for proper training of radiologists and scholarly research in the field of radiology.

#### Equipment and infrastructure

Modern equipment and infrastructure are crucial to the training of competent subspecialist radiologists. The poor funding of health care in many African nations has made it impossible to acquire new equipment. These inadequacies impede the creation of the top-level educational environment required for specialist and subspecialist tutoring.^63^

#### Expertise

The quality of experts in a field is a product of the training that the specialists had received. It takes a subspecialist to train a subspecialist. Currently, many African countries have insufficient subspecialist manpower, which makes it difficult to initiate subspecialisation training programmes.

#### Emigration

Many African medical graduates aim to leave the continent for greener pastures abroad. This medical exodus robs the continent of critically needed expertise.^64,65^ The mass emigration further worsens the already low radiologist-to-population ratio in many African countries.

African radiologists seeking subspecialisation often travel outside Africa to train, but many of them do not return to their home country once they find better opportunities abroad. If subspecialisation programmes are established locally, the attrition of African radiologists might be mitigated.^66,67^

#### Politics

Sometimes, obtaining approval for new programmes requires savvy political manoeuvring, lobbying, and horse-trading. Bureaucratic red tape is a feature of governmental regulatory agencies worldwide, and African nations are no different. Radiology is a relatively ‘invisible’/‘behind-the-scenes’ medical speciality. Consequently, it might be an uphill task to secure governmental backing and funding for programmes without accruable political mileage.^68^ Interprofessional rivalries at the universities and teaching hospitals could also derail the successful take-off of new training programmes.^69^

### Recommendations

Given the perennial obstacles to subspecialisation enumerated above, especially funding constraints, African radiologists (with foreign collaboration when needed) can leverage existing and emerging technologies and innovative training/learning methods to streamline the training of subspecialist radiologists.

Web-based training was piloted in Ethiopia over a 20-month period using pre-recorded online lectures, case reviews, and learning modules, overseen by subspeciality-trained radiologists of the Johns Hopkins University School of Medicine.^21^ A similar web-based learning tool for paediatric radiology (with users all over the United States and 53 other countries) is used to deliver paediatric radiology curriculum internationally by paediatric radiologists in the USA.^70,71^ Web-based training is cheaper, adaptable, more accessible, and effective.^21,72^

The European diploma in emergency radiology subspeciality is delivered using a combination of self-directed learning, webinars, workshops, research & teaching, as well as on-the-job training.^73^ Electronic teaching files and internet-accessible case collections are also being used increasingly for radiology subspecialisation training in other European countries with limited access to complex equipment.^25^

Simulation-based technology and immersive training environment for IR have also been proposed.^74,75^

A complementary business model of medical subspeciality training that incorporates the private sector has been developed for reproductive medicine in South Africa by Dalmeyer et al.^76,77^ The authors are convinced that the model can be applied to other subspecialities.

## Conclusion

There is limited availability of subspecialist radiology training in African countries. Cost-effective and innovative approaches to training are needed to address this deficit.

This study was limited by unavailability of data on subspecialisation in Algeria, Libya, and Senegal.
