# Corrigendum: Radiology subspecialisation in Africa: A review of the current status

**DOI:** 10.4102/sajr.v26i1.2347

**Published:** 2022-06-30

**Authors:** Efosa P. Iyawe, Bukunmi M. Idowu, Olasubomi J. Omoleye

**Affiliations:** 1 College of Medicine, University of Ibadan, Ibadan, Oyo State, Nigeria; 2 Department of Radiology, Union Diagnostics and Clinical Services PLC, Yaba, Lagos State, Nigeria; 3 Department of General Medicine, LouisMed Hospital and Fertility Centre, Lekki Phase 1, Lagos State, Nigeria

In the published article, Iyawe EP, Idowu BM, Omoleye OJ. Radiology subspecialisation in Africa: A review of the current status. S Afr J Rad. 2021;25(1):a2168. https://doi.org/10.4102/sajr.v25i1.2168, on page 4 the following paragraph is updated as it was incorrectly formulated:

The original incorrect wording:

The Tanzanian IR project was midwifed by the RAD-AID International IR programme in collaboration with academic institutions in the United States and Europe.

The revised and updated wording:

The Tanzanian IR project was midwifed by the Radiological Society of North America (RSNA) and an organisation called Road2IR (www.road2ir.org).

The authors apologise for this error. The correction does not change the significance of study’s findings or overall interpretation of its results or the scientific conclusions in any way.

